# Study on Bending Creep Performance of GFRP-Reinforced PVC-Based Wood-Plastic Composite Panels

**DOI:** 10.3390/polym14224789

**Published:** 2022-11-08

**Authors:** Bangbang Dai, Ruili Huo, Kun Wang, Zhengqing Ma, Hai Fang

**Affiliations:** College of Civil Engineering, Nanjing Tech University, Nanjing 211816, China

**Keywords:** GFRP reinforced, wood-plastic composites, bending creep, load level, four-element model

## Abstract

Wood-plastic composites (WPCs) are environment-friendly materials, which have broad application prospects in structures. They cannot be used for bearing structures because of poor mechanical performance and creep deformation. In order to enhance the mechanical behavior and decrease the long-term creep deformation, glass fiber reinforced plastics (GFRP) sheets and rebar reinforcement design methods are proposed. The bending static tests and creep performance tests of WPCs were conducted. The results showed that GFRP sheets and rebars improved the ultimate flexural loading capacity and deformation capacity by 257% and 165%, respectively, decreased the creep deflection effectively, and avoided shear failure. When the load level was very low, the creep deformation of WPC panels unreinforced, or reinforcement developed stably with time, and the damage did not occur within 1100 h. When the load increased to 80% of the ultimate load level, all specimens were damaged in the compression zone, the creep deformation increased quickly and unstably, bending shear failure of the unreinforced specimen occurred after 7 h, shear failure of the GFRP-sheets-reinforced specimen occurred after 1100 h, and the rebar-reinforced specimen failed after 720 h with excessive deflection deformation in the span. The reinforced effect of GFRP sheets is better. The creep strain growth rate of all specimens increased quickly at the first stage and gradually decreased at the second stage and tended to be stable. The creep calculation model was built based on the four-element model, which is simple and efficient and can make scientific and reasonable predictions of the two phases of structural transient creep and deceleration creep.

## 1. Introduction

Composite material structures are more popular in engineering applications due to their excellent physical and mechanical properties, especially under harsh environmental conditions [1,2,3]. PVC-based wood-plastic composites are a new type of environmentally friendly composite material made from wood fibers or plant fibers as reinforcing materials or fillers and polyvinyl chloride thermoplastics as the matrix, which are melt-mixed and then subjected to a molding and processing process [4]. They have the advantages of good processing performance, corrosion resistance and mechanical performance, wide resource of raw materials, energy saving and environmental protection, weather resistance, creep performance is higher than natural wood [5], and they have been widely used as structural materials in the field of garden architecture, exterior wall panels, outdoor decking, indoor and outdoor decoration, automotive interior trim, etc. However, WPCs are deficient in weather resistance, flame retardancy, mechanical strength, etc., especially their poor creep resistance, which is prominent in long-term use, affecting their application in the engineering field. GFRP has the advantages of high tensile strength, high modulus of elasticity, corrosion resistance, abrasion resistance, and low cost, which is also used to reinforce resin synthetic composites [6,7,8].

In recent years, researchers have conducted studies on the component materials’ behavior [9,10,11,12], manufacture processes [13,14], structural forms [15,16,17], and mechanical performance [18,19,20,21] of WPCs. As the resin material exhibits viscoelastic properties, the resin molecules are displaced along the direction of the stress field under continuous loading, and the molecular chains are gradually straightened from their original bent state to result in creep [22,23]. GFRP creep is mainly divided into the following processes: ① local elongation of glass fibers; the resin has a restraining effect on the fiber; ② local fracture of glass fibers; the resin around the fiber transfers stress, and the resin creep in the high-stress area increases; ③ when the shear stress at the fiber resin interface is parallel to the fiber filaments, the interface damage occurs in single fiber bundles under the effect of continuous stress, and combined with the effect of their own defects, leads to the development of cracks; ④ a number of fiber bundle fractures; the creep rate keeps increasing [24,25,26].

Results have been obtained on the creep properties of GFRP under long-term loading. Dutta et al. [27] studied the tensile and compressive properties of GFRP rebars under continuous loading and high temperature, analyzed the effect of the superposition effect of temperature and time on the creep properties of GFRP rebars, and proposed a simple empirical model to predict the failure time of GFRP based on the equation fitted to the creep curve. Tannous et al. [28] found that the creep resistance is better and the deformation is smaller when the GFRP rebars are at consistently lower stress levels. Uomoto et al. [29] found that the creep deformation of GFRP rebars is large when the sustained stress level reaches 80% of the ultimate strength of the GFRP rebars. Guowei Li et al. [30,31] found that GFRP creep occurs only when the load level reaches 77% of the ultimate load, but creep deformation of GFRP was not found in the experiment, indicating that GFRP has excellent creep resistance under the effect of a long-term low load level, which is consistent with the results of Qi Sun [32]. Yihua Cui et al. [33] concluded that the interaction of glass fiber, wood powder, and plastic formed a stable three-dimensional spatial structure, which existed in the plastic matrix as a strong skeleton and prevented the generation and expansion of cracks, thus improving the mechanical strength and impact resistance of the composites; the mechanical properties of WPCs show a trend of increasing and then decreasing with an increase in glass fiber content. Dong Guo et al. [34] found that the impact strength, flexural strength, and flexural modulus of the composites reached the maximum when the content of glass fiber was 30%, which were 121%, 174%, and 79% higher than those of the composites without glass fiber, respectively; the mechanical properties decreased when the glass fiber content increased to 40%. Jeamtrakull et al. [35] showed that the wear resistance of the composites was significantly improved by the addition of 10% glass fibers (length 3–13 mm), indicating that the addition of short-cut glass fibers could improve the wear resistance of the composites. Huang and Kim [36,37] prepared WPCs with a core/shell structure by co-extrusion, using short-cut glass fiber-reinforced high-density polyethylene for the shell and high-density polyethylene WPCs for the core. The results showed that the fibers in the shell reduced the linear expansion of the material, and the shell thickness and fiber content had different reinforcing effects on the core WPCs. Overall, the core/shell structure reduces the amount of fiber and achieves reinforcement at the same time. Zolfaghari et al. [38] developed WPCs reinforced with continuous untwisted glass fiber bundles and showed that the continuous glass fiber-reinforced WPCs have a better reinforcement effect than short-cut fibers, which is significant to solve the brittleness problem and expand the applications of WPCs. Pulngern et al. [39,40] investigated the mechanical performance of WPCs under different temperatures and loads; Sain et al. [41] improved the existing creep model and Findley index model for WPCs; Li et al. [42] investigated the flexural creep performance of glass fiber-reinforced polymer composite sandwich beams and proposed a life prediction method to predict the creep life of beams under arbitrary load levels. Xianling Tian et al. [43] studied the creep performance of WPCs under different loading ways; Yan Cao et al. [44] investigated the creep performance and creep model of molded poplar wood fiber (PWF)/HDPE composites; Huhu Du et al. [45] and Weiren Xie [46] simulated the creep behavior of WPCs using the multi-element model and power law model based on experimental results. Some technical standards were made through experimental studies on the creep performance of WPCs [47]. Some scholars have studied the effects of material factors, process factors, environmental factors, and service factors on the creep performance of WPCs [48,49,50,51,52]. Conventional composite components are highly susceptible to peeling damage at the interface between the face and core during fabrication and in service, which severely limits their lightweight and high-strength properties [53,54,55]. The creep deformation caused by long-term loading cannot be ignored when WPCs are used as structural members under constant environmental and load effects, and the creep deformation will lead to the reduction of structural bearing capacity or instability damage, and the damage caused by creep is one of the main forms of damage to the members, which directly affects the reliability and safety of the structure, so it is mainly used in the field of non-structural materials [56]. Most materials’ creep will occur under long-term loading [57,58,59], which causes material, structural strength, and stiffness degradation [60], affecting the safety, serviceability, and durability of structures. GFRP has the characteristics of a light weight and high strength, corrosion resistance, fatigue resistance, and excellent creep resistance [61,62,63]. This paper used the GFRP reinforcement method to overcome the shortcomings of PVC-based WPCs, such as insufficient mechanical properties and large creep deformation, and carried out three-point bending creep performance tests under different load levels to provide theoretical references for the design of WPCs’ loading structural members and improve the durability performance of structures.

## 2. Experiments

### 2.1. Raw Materials

Based on the application of WPCs, we chose the widely used PVC-based WPC materials to make specimens. The density was 0.82 g/cm^3^, Young’s modulus was 960 MPa, bulk modulus of elasticity was 533 MPa, yield strength was 9.7 MPa, and Poisson’s ratio was 0.2, which were produced by Keju New Material Technology Co, Ltd. Anhui, China. The main components were poplar wood powder, PVC resin, foaming agent, coupling agent, etc., prepared by the co-extrusion molding process. The tensile strength of the GFRP sheet was 300 MPa, the modulus of elasticity was 20 GPa, Poisson’s ratio was 0.15; the tensile strength of the GFRP rebar was 490 MPa and the modulus of elasticity was 42 GPa.

### 2.2. Samples’ Design and Preparation

**Figure 1:**
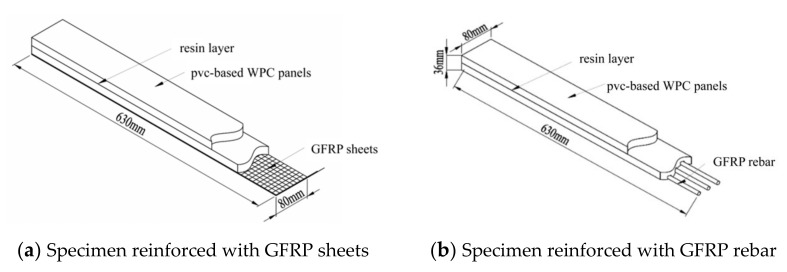
Design of GFRP-sheets- and rebar-reinforced WPCs.

**Figure 2:**
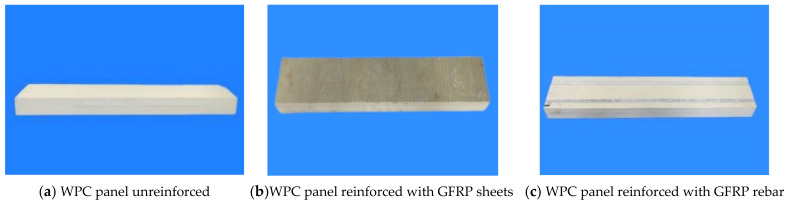
PVC-based WPC specimens.

There are three types of specimens: WPCs, GFRP-sheets-reinforced WPCs, and GFRP-rebar-reinforced WPCs. The dimensions of specimens were: 630 mm × 80 mm × 36 mm, as shown in Figure 1. GFRP-sheets-reinforced WPCs were made of a double-layer PVC-based wood-plastic panel and GFRP sheet; the thickness of the GFRP sheet was 1.2 mm, which consists of an unsaturated polyester resin matrix and biaxially orthogonal (0, 90°) woven glass fiber cloth in one piece produced by vacuum introduction technology [64] and two layers of 0.6 mm GFRP sheets glued together. The PVC-based wood-plastic panels were cut according to the designed size and a margin of 10 mm was left on the length and width to facilitate the secondary processing of the specimen after the gluing was completed. Considering that the PVC-based WPC panels’ surface was too smooth and affected the gluing quality during production, the polishing process was necessary. The specimens were cut after 48 h of curing. The GFRP-rebar-reinforced PVC-based wood-plastic panels were prepared by an embedding process, and the diameter of the GFRP threaded rebar was 8 mm. Firstly, longitudinal penetration grooves were engraved on the bottom surfaces of WPCs, then epoxy resin glue was filled in the groove, and the GFRP rebars were embedded in the opened groove, as shown in Figure 2.

### 2.3. Bending Static Load Test

Firstly, the three-point bending static load test of the reinforced and unreinforced specimens was carried out to determine their ultimate load capacity and in order to determine the load level of the bending creep performance test. The span sizes of the specimens were 576 mm, an MTS electronic universal testing machine was used in the test, and the loading rate was 2 mm/min by the method of controlled displacement. In order to prevent the local damage of the specimen surface during the loading process, a rubber mat of Shore A hardness 60 and size of 70 mm × 25 mm × 3 mm was placed at the span loading point on the upper surface of the specimen. The load-mid-span displacement curves were collected and recorded simultaneously during the loading process. 

### 2.4. Bending Creep Test

**Figure 3:**
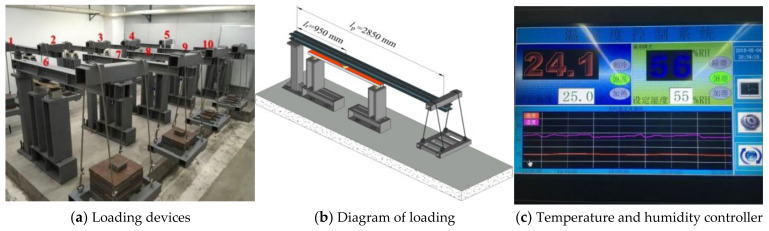
Three-point bending creep test loading devices.

The bending creep test was carried out under constant temperature and humidity with three-point loading; the test device is as shown in Figure 3. The temperature and humidity controller could provide a stable temperature and humidity environment for the creep test; the temperature was kept at 23 °C ± 2 °C, the relative humidity was 50% ± 10%, and the displacement of the specimen was measured by dial indicator with the accuracy of 0.01 mm.

Six load levels were set as the creep test design load: 30%, 40%, 50%, 60%, 70%, and 80% of the ultimate bearing capacity of the static test. The rubber mat was placed on the upper surface of the test piece span, adjusting the position of the dial indicator, so that its pointer was perpendicular to the lower surface of the specimen. When the loading end slowly reached the position of the rubber mat, the initial reading of the dial indicator was recorded. After that, the data were collected every 24 h. The test time for each specimen was set to 1800 h.

## 3. Results and Analysis

### 3.1. Results and Analysis of Bending Static Load Test

**Figure 4:**
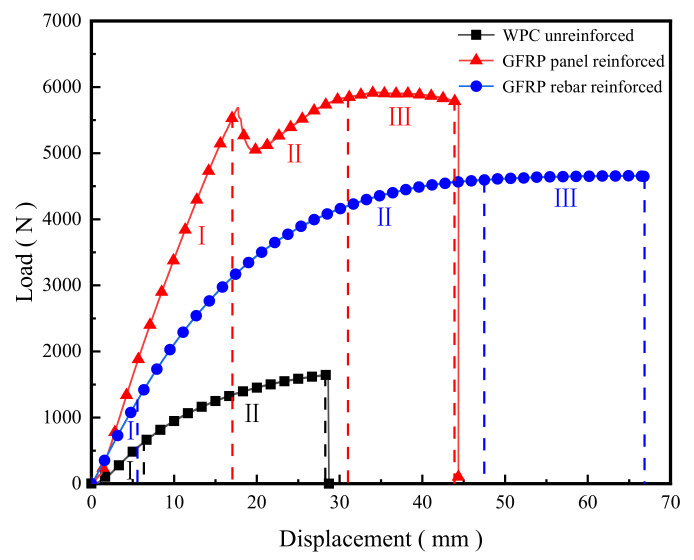
Load-mid-span displacement curves of WPC panels in static load test.

The load-displacement curves are shown in Figure 4; the mid-span displacements of the three specimens show three stages of deformation characteristics, which are elastic stage I, elastic–plastic stage II, and plastic stage III. The loads of the three specimens in the elastic stage Ⅰ grew in proportion to each other at the same displacement, and the elastic stage of the GFRP-sheets-reinforced specimen was the longest. The GFRP-sheets-reinforced WPC specimen reached elastic–plastic stage II when the span displacement increased to 15 mm, showing a small decrease in load while the span displacement increases quickly, fluctuated in a small range, and then the specimen yielded and reached the elastic–plastic stage II, with a displacement of 30 mm to reach the plastic stage Ⅲ. At this time, the mid-span displacement reached 44.37 mm, which was the second largest in these specimens, then shear failure occurred. The GFRP-rebar-reinforced specimen reached the elastic–plastic stage when the mid-span displacement was about 5 mm, which is almost the same as the unreinforced specimen. The displacement gradually increased during the loading process; when the load gradually became stable, the specimen had the obvious plasticity of stage Ⅲ, and the displacement still increased. The GFRP-rebar-reinforced specimen had the best ductility and the largest displacement of 66.77 mm.

The results of the three-point bending loading test are shown in Table 1. The bearing capacity and ductility of the two GFRP-reinforced specimens are greatly increased. Compared with the unreinforced specimen, the average ultimate bearing capacity of the GFRP-sheets-reinforced specimens was increased by 258% and the mid-span displacement was increased by 55%; the average ultimate bearing capacity of the GFRP-rebar-reinforced specimens was increased by 165% and the mid-span displacement was increased by 133%.

### 3.2. Results and Analysis of Bending Creep Test

#### 3.2.1. Failure Mode

**Figure 5:**
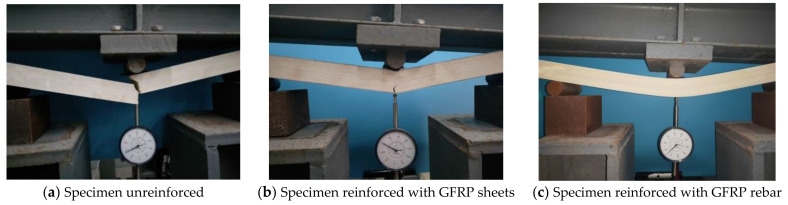
Failure mode of PVC-based WPC specimens.

During the bending creep test, failure did not occur under low load levels. The mid-span deflection of the specimen increased significantly during the transient loading, and with the increase in time, the creep deflection of each specimen increased slowly and stabilized, which showed two stages of typical creep, transient creep and deceleration creep, and none of them reached the accelerated creep damage stage. Shear failure occurred in unreinforced specimens at the 80% load level. At the instant of loading, the mid-span deflection of the specimen increased rapidly accompanied by a slight resin fracture sound. With the loading time increasing, many small cracks appeared on the lower surface of the specimens after 1 h, the cracks kept extending upward, and they finally fractured after 7 h, which is a typical brittle failure, as shown in Figure 5a. The movement of molecular chains mainly occurs in the creep process of polymer materials. In the transient creep phase, the bond lengths and bond angles of molecular chains are changed due to the loading effect, and the curled chains are unfolded. When the unfolded chains are restrained, the deceleration creep phase is entered and the slip between different molecular chains occurs. The polymer matrix plays the role of bonding wood fibers and transferring stress inside the wood-plastic material, while the polymer molecular chain movement is restrained to some extent by the wood fibers. The GFRP-sheets-reinforced specimens made a slight sound of resin and fiber fracture during loading at the 80% load level, the GFRP sheets were folded and whitened, and the deflection deformation in the span increased significantly. A small crack appeared on the specimen after 840 h from the bottom across the middle and gradually extended, and the glass fibers were pulled off and bending shear damage occurred after 960 h; the crack suddenly appeared as shown in Figure 5b. The GFRP-rebar-reinforced specimens were considered to fail due to excessive deformation of their mid-span deflection at the higher load levels of 70% and 80%; there were no cracks or fractures, and the mid-span deflection increased significantly, as shown in Figure 5c. It can be seen that the shear resistance of the WPC specimens reinforced by GFRP sheets was improved, and the ultimate loading capacity of the specimens was significantly increased. The bending resistance of the WPC specimens reinforced by the GFRP rebar was improved, which showed better ductility and interfacial properties. The failure mode was also changed from brittle shear failure to bending shear damage or excessive mid-span deformation.

#### 3.2.2. Creep Strain–Time Curves

**Figure 6:**
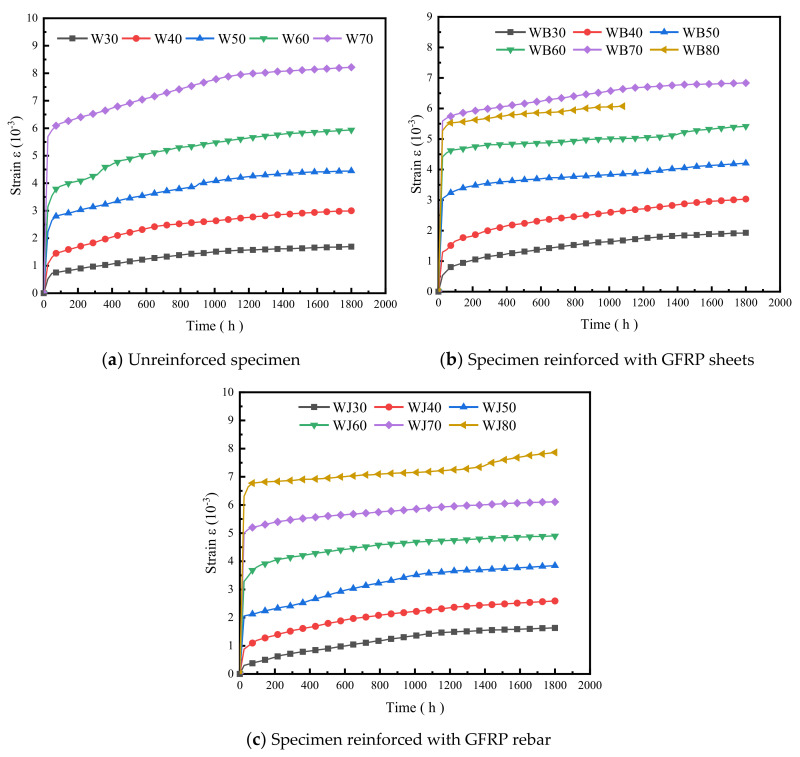
Bending creep strain–time curve of PVC-based WPCs (W represents the unreinforced WPC specimen type, WB represents the type reinforced with GFRP sheets, and WJ represents the type reinforced with GFRP rebar).

Figure 6 shows the bending creep strain–time curves of WPC panels. It can be seen from Figure 6a that the strains of the unreinforced specimens gradually increased with time. The final strain rates grow by 226.67%, 182.11%, 99.61%, 88.74%, and 44.31% under the five stress levels from 30% to 70%, respectively. From Figure 6b, the final strain growth rates of the GFRP-sheet-reinforced specimens are 259.77%, 134.00%, 37.96%, 22.70%, 22.26%, and 15.25% from 30% to 80%. From Figure 6c, the final strain growth rates of the GFRP-rebar-reinforced specimens are 440.00%, 194.12%, 86.97%, 49.21%, 22.11%, and 24.66% from 30% to 80%. The initial creep strains of the three types of specimens are transient and similar. Transient deformation occurred when the designed load was carried out at different load levels, and the initial strains increased with the increase in the load level. The development trend of the initial strains of the specimens at the same load level is similar. It can be seen from the figures that the creep strains of the specimens under low load levels are relatively close, the creep deformation increases with time, the deformation rate declines gradually, and the overall creep development is relatively smooth. The creep strain of the specimens at high load levels grows rapidly and the deformation rate is significantly larger than that at low load levels.

As shown in Figure 6a, after the initial quick increasing stage, the creep strain of the unreinforced specimens gradually increases with time and the strain rate shows a deceleration trend. With the increase in the load level, the strain rate increases. Shear failure occurred rapidly after 7 h loading at the 80% load level. The time is so short that we cannot see this in Figure 6a, while the specimens under other load levels reach the decelerating creep stage and stabilize after the transient creep stage.

The strain of the GFRP-sheets-reinforced specimens is shown in Figure 6b; cracks failure occurred after 1000 h at the 80% load level, which is in the creep stabilization stage. The strain almost kept stable with time after 1200 h at the lower load level. Figure 6c represents that the initial strains of the GFRP-rebar-reinforced specimens are largest at the 80% load level, the failure did not occur but the strain rate increases significantly with time, and the strain should not be negligible at the last stage.

**Figure 7:**
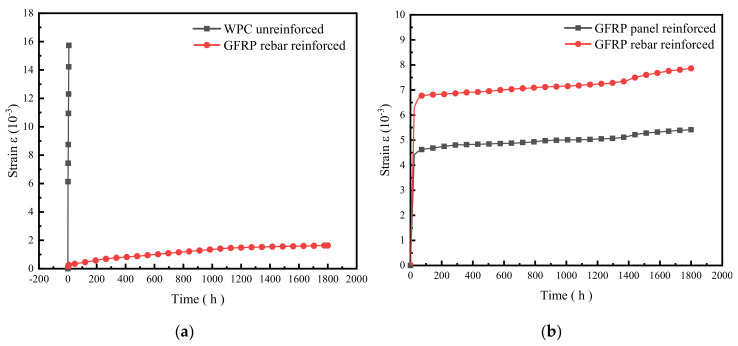
Comparison of bending creep strain–time curves of unreinforced and reinforced WPCs at the same load. (**a**) Strain comparison between unreinforced and reinforced with GFRP rebar specimens (load 1400 N). (**b**) Strain comparison between GFRP-sheets- and rebar-reinforced specimens (load 3600 N).

Figure 7 shows the bending creep strain–time curves of the three types of specimens at the same loads. As shown in Figure 7a, the bending creep curves of unreinforced and GFRP-reinforced specimens at the same load of 1400 N are significantly different; the unreinforced specimen shows a significant increase in strain during transient loading and a non-stationary rapidly increasing strain rate during 7 h loading. The strain of GFRP-rebar-reinforced specimens increased during the transient loading and gradually increased during the loading process, the strain rate gradually decreased and became stable, and creep development was relatively smooth overall. The final strain value of the unreinforced specimen was 960% higher than that of the GFRP-rebar-reinforced specimen; the GFRP-rebar-reinforced method not only improved the load capacity of WPCs but also reduced the creep strain effectively.

Figure 7b demonstrates the bending creep curves of GFRP-sheets- and rebar-reinforced specimens at the same load of 3600 N; the change trend is similar in shape, both strains increased rapidly during transient loading and the strain tended to be stable after 72 h, the strain rate gradually decreased, and the overall creep strain development was relatively smooth. The final strain value of GFRP-rebar-reinforced specimens was 150% higher than that of the GFRP-sheets-reinforced specimens. With the increase in time, the strain value of the GFRP-sheets-reinforced specimens was always smaller than that of GFRP-rebar-reinforced specimens, which indicated that the former had stronger bending resistance and a better reinforcement effect than the latter.

Overall, at the same load, the creep resistance of the GFRP-sheets-reinforced specimen is the strongest and the reinforcement effect is the best, and the creep resistance of the GFRP-rebar-reinforced specimens is much better than that of the unreinforced specimens. The best reinforcement method to reduce bending creep deformation is reinforcement with GFRP sheets.

In order to study the growth law of creep strain with time variation at different load levels and to evaluate the variation law of creep more reasonably, the definition of relative creep from ASTM D6815-09 [65] is:

(1) $$\zeta =\varepsilon_{t}/\varepsilon_{o}$$

where ζ is the relative creep of the strain at time *t* relative to the initial strain; $\varepsilon_{t}$ is the strain at time *t*; and $\varepsilon_{o}$ is the initial strain.

**Figure 8:**
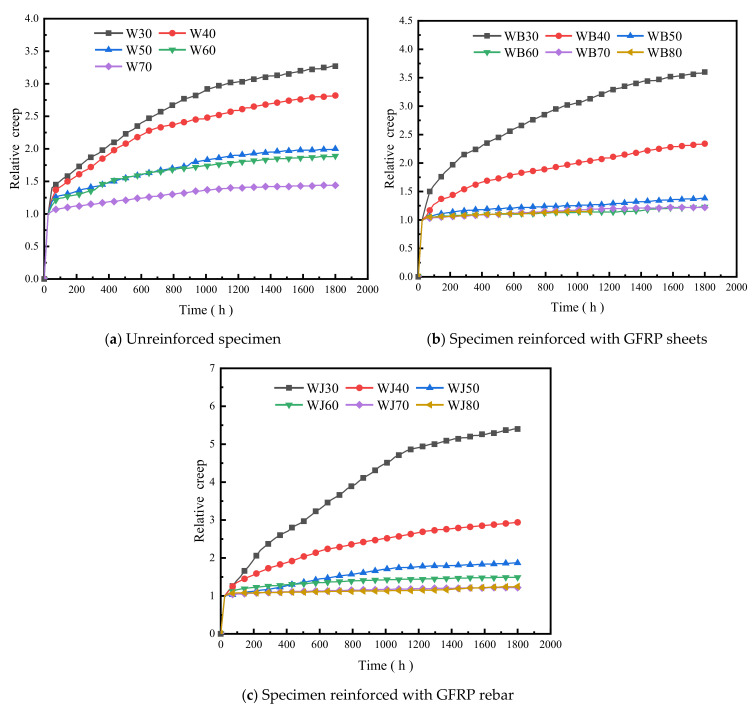
Relative creep–time curve of PVC-based WPCs.

The test data were processed uniformly to obtain the relative creep–time curves of the three specimen types, as shown in Figure 8. The curves show that the value of relative creep increases with time. The final relative creep of the unreinforced specimens was 327%, 282%, 199%, 189%, and 144% of the initial values under each load level from 30% to 70%; the GFRP-sheets-reinforced specimens were 360%, 234%, 138%, 123%, 122%, and 121% of the initial values from 30% to 80%; and the GFRP-rebar-reinforced specimens were 540%, 294%, 187%, 149%, 122%, and 122% of the initial values from 30% to 80%. The relative creep growth rate of the three specimen types was generally decelerated, indicating that the second stage of creep of wood-plastic specimens had a decelerated creep stage. Since the initial strain value is larger for specimens with a high load level under transient loading, the strain value changes gently during the deceleration creep phase, so the relative creep decreases with the increase in load level. The slope of the curve differs for specimens with different load levels. For the specimens of the same type, the higher the load level, the smaller the relative creep value and the slower the growth at a later stage. The relative creep variation trends of the three types of members are closer when the load rating is higher.

## 4. Four-Element Theoretical Model

**Figure 9:**
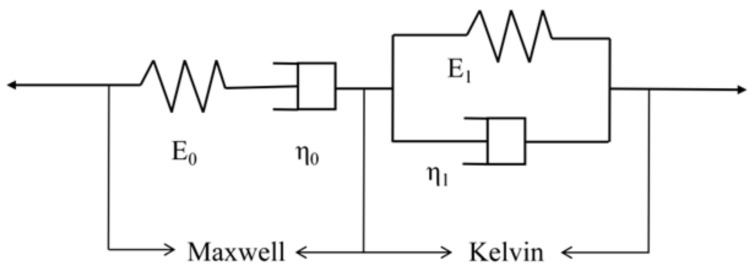
Four-element model.

Based on the above experiment analysis, the bending creep behavior of the specimen is consistent with the four-component model, which describes the development law of viscoelastic creep, which consists of one spring unit, one Kelvin–Voigt model, and one viscous pot unit in series, as shown in Figure 9, which is used to describe the deformation characteristics of the three parts of the material: normal elastic deformation, high elastic deformation, and viscous flow, respectively.

The generic expression for the model is:

(2) $$\varepsilon \left(t\right)=\frac{\sigma }{E_{0}}+\frac{\sigma_{t}}{\eta_{0}}+\frac{\sigma }{E_{1}}\left[1-\exp\left(-tE_{1}/\eta_{1}\right)\right]$$

where *t* is time; *ε*(*t*) is the total material strain at time *t*; *σ* is the constant stress; $E_{0}$ is the material modulus of elasticity; $\eta_{0}$ is the material viscosity coefficient; $E_{1}$ is the elasticity coefficient; and $\eta_{1}$ is the viscosity coefficient.

**Figure 10:**
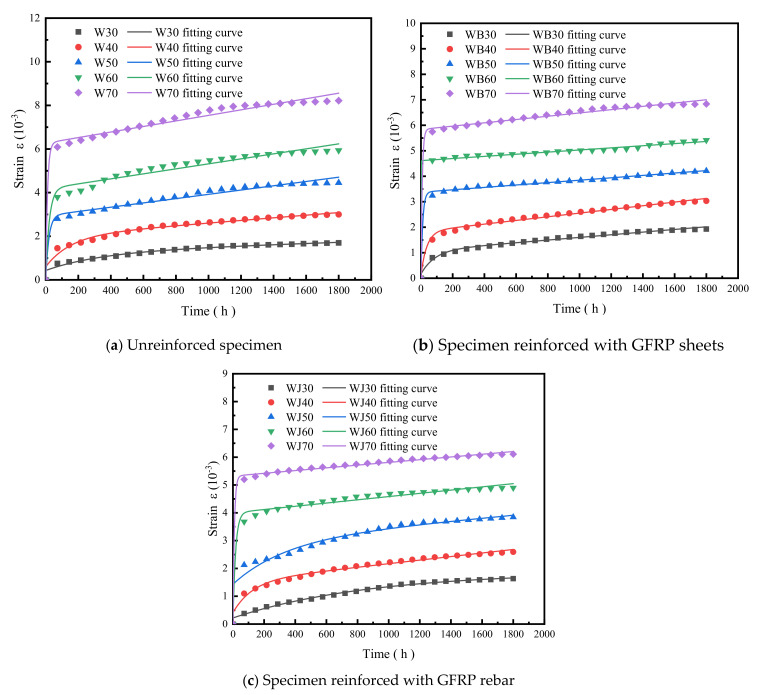
Fitting curve of four-element model of PVC-based WPCs.

The model equation can represent the three components of material creep: $\sigma /E_{0}$ for elastic deformation, $\sigma_{t}/\eta_{0}$ for viscous deformation, and $\left(\sigma /E_{1}\right)\left[1-\exp\left(-tE_{1}/\eta_{1}\right)\right]$ for viscoelastic deformation. It is known from the deformation element that the viscous creep of the model increases with time if the viscosity coefficient $\eta_{1}$ is determined. The model was used to fit and analyze the bending creep test results of PVC wood-plastic composites to obtain the creep strain–time curve, as shown in Figure 10.

Figure 10 shows that the fitted curves of the four-element model are closer to the test results under low load levels and the correlation coefficients of the curve fits are larger than 0.97, with good fitting accuracy. The fitting error of unreinforced specimens increases slightly and the strain rate increases as the load level increases; the correlation coefficients of the fitted curves for both GFRP-sheets- and rebar-reinforced specimens are high for each load level, which shows that the four-element model is suitable for predictive fitting analysis of creep strain in two stages of transient creep and deceleration creep of WPCs and can be used for long-term creep fitting analysis and life prediction of creep in the first two stages.

## 5. Conclusions

GFRP-sheets- and GFRP-rebar-reinforced specimens were designed to improve the bearing capacity and reduce the bending creep performance of WPCs, the static experiments and long-time bending creep experiments were carried out, and creep strain prediction analysis was accomplished based on the four-element model. The conclusions are as follows:

(1) The ultimate load-bearing capacity of WPC panels is greatly improved with GFRP sheets and rebar reinforcement, and the ultimate bearing capacity of the GFRP-sheets-reinforced specimen increased by 258% and the GFRP-rebar-reinforced specimen increased by 165%. Using GFRP to reinforce WPCs is effective.
(2) The failure mode of PVC-based WPCs is closely related to the load level and the reinforcement method. After the transient stage, the creep kept decreasing and developed stably without damage under the low load level. When the loading level reached 80% of the ultimate load, unreinforced specimens suffered shear failure after 7 h, the maximum deflection in the span was 19.10 mm. The mid-span deflection of the GFRP-sheets-reinforced specimen increased significantly under transient loading, the GFRP sheets’ fibers were pulled out and bending shear failure occurred after 960 h, and the maximum deflection in the span was 7.03 mm. GFRP-rebar-reinforced specimens had significant deformation and were considered to fail due to their excessive mid-span deflection deformation, no cracks or fractures appeared, and the specimens had excellent ductility.
(3) The creep strain change rules of specimens were obtained under different load levels. The strain increased and the strain growth rate decreased with time. The final strain rates of the unreinforced specimen increased by 226.67%, 182.11%, 99.61%, 88.74%, and 44.31% under the loading levels from 30% to 70%; those of the GFRP-sheets-reinforced specimen were 259.77%, 134.00%, 37.96%, 22.70%, 22.26%, and 15.25% under the loading levels from 30% to 80%; those of the GFRP-rebar-reinforced specimen were 440.00%, 194.12%, 86.97%, 49.21%, 22.11%, and 24.66% under the loading levels from 30% to 80%. The strain growth rates of the three types of specimens are generally decelerated. The relative creep decreased with increasing load levels. The final relative creep of unreinforced specimens was 327%, 282%, 199%, 189%, and 144% under the load levels from 30% to 70%; that of the GFRP-sheets-reinforced specimens was 360%, 234%, 138%, 123%, 122%, and 121%; and that of the GFRP-rebar-reinforced specimens was 540%, 294%, 187%, 149%, 122%, and 122% under the load levels from 30% to 80%.
(4) Based on the test results, a four-element model was built. It is a simple, accurate, and reasonable classical model for describing the creep performance of WPC either reinforced by GFRP or not. The model can be used for long-term strain analysis and life prediction of specimens in the first two stages of transient creep and deceleration creep. GFRP-reinforced design methods can effectively improve the mechanical properties and creep resistance of WPCs and broaden the application field of WPC products.

## Figures and Tables

**Table 1:** Static load test results of three-point bending for WPCs.

Specimen Types	Failure Mode	Specimen Number	Ultimate Load (N)	The Average (N)	Mean Mid-Span Displacement (mm)	The Average (mm)
Unreinforced	Shear failure	1	1738	1720	27.62	28.63
		2	1715		28.46	
		3	1707		29.81	
Reinforced with GFRP sheet	Bending shear failure	1	6165	6150	43.24	44.30
		2	6151		44.12	
		3	6134		45.75	
Reinforced with GFRP rebar	Exceed displacement limit	1	4576	4560	65.22	66.70
		2	4558		65.86	
		3	4546		69.23	

## Data Availability

The data presented in this study are available on request from the corresponding author.

## References

[1] Alrubaie M.A.A., Lopez-Anido R.A., Gardner D.J. (2020). Flexural creep behavior of high-density polyethylene lumber and wood plastic composite lumber made from thermally modified wood. Polymers.

[2] Abdullah A., Emin S., Hayrettin D., Ömer S.S., Mürsel E. (2021). The effects of harsh aging environments on the properties of neat and MWCNT reinforced epoxy resins. Constr. Build. Mater..

[3] Abouelregal A.E., Marin M. (2020). The size-dependent thermoelastic vibrations of nanobeams subjected to harmonic excitation and rectified sine wave heating. Mathematics.

[4] Wang Q.W., Wang W.H. (2007). Wood-Plastic Composites and Products.

[5] Wang D.Z., Zhu M. (2019). Research progress of biomass WPCs. Sci. Technol. Innov..

[6] Li C., Xian G., Li H. (2019). Effect of postcuring immersed in water under hydraulic pressure on fatigue performance of large- diameter pultruded carbon/glass hybrid rod. Fatigue Fract. Eng. Mater..

[7] Lal H.M., Uthaman A., Li C.G., Xian G.J., Thomas S. (2022). Combined effects of cyclic/sustained bending loading and water immersion on the interface shear strength of carbon/glass fiber reinforced polymer hybrid rods for bridge cable. Constr. Build. Mater..

[8] Pan Y.F., Yan D.M. (2021). Study on the durability of GFRP bars and carbon/glass hybrid fiber reinforced polymer (HFRP) bars aged in alkaline solution. Compos. Struct..

[9] Bledzki K., Andrzej, Omar F. (2004). Creep and impact properties of wood fibre-polypropylene composites: Influence of temperature and moisture content composites. Sci. Technol..

[10] Adrian J., Marcovich E. (2004). Norma Analysis of the Creep Behavior of Poly propylene-Woodfiber Composites. Polym. Eng. Sci..

[11] Lee S.Y., Yang H.S., Kim H.J., Jeong C.S., Lim B.S., Lee J.N. (2004). Creep behavior and manufacturing parameters of wood fiber filled poly propylene composites. Compos. Struct..

[12] Hung K.C., Wu L.T., Chen Y.L. (2016). Assessing the effect of wood acetylation on mechanical properties and extended creep behavior of wood/recycled-polypropylene composites. Constr. Build. Mater..

[13] Pooler D.J., Smith L.V. (2004). Nonlinear Viscoelastic Response of a Wood- Plastic Composite Including Temperature Effects. J. Thermoplast. Compos..

[14] Park P.D., Balatinecz J.J. (2004). Short term flexural creep behavior of wood- fiber/polypropylene composites. Polym. Compos..

[15] Fang H., Sun H., Liu W., Wang L., Bai Y., Hui D. (2015). Mechanical performance of innovative GFRP-bamboo-wood sandwich beams: Experimental and modelling investigation. Compos. Part B Eng..

[16] Tuwair H., Hopkins M., Volz J., Eigawady M.A., Mohamed M., Chandrashekhara K., Birman V. (2015). Evaluation of sandwich panels with various polyurethane foam-cores and ribs. Compos. Part B Eng..

[17] Shi H., Liu W., Fang H., Bai Y., Hui D. (2017). Flexural responses and pseudo-ductile performance of lattice-web reinforced GFRP-wood sandwich beams. Compos. Part B Eng..

[18] Gigliotti L., Pinho S.T. (2016). Prediction of the post-crushing compressive response of progressively crushable sandwich foam cores. Compos. Part A Appl. Sci..

[19] Zhang P., Cheng Y., Liu J., Li Y., Zhang C., Hou H., Wang C. (2016). Experimental study on the dynamic response of foam-fifilled corrugated core sandwich panels subjected to air blast loading. Compos. Part B Eng..

[20] Chen Q., Linghu T., Gao Y., Wang Z., Liu Y., Du R., Zhao G. (2017). Mechanical properties in glass fiber PVC-foam sandwich structures from different chopped fiber interfacial reinforcement through vacuum-assisted resin transfer molding (VARTM) processing. Compos. Sci. Technol..

[21] Badaruzzaman W.H.W., Dabbagh N.M.R., Salleh K.M., Saharuddin E.N., Radzi N.F.M., Azham M.A.A., Sani S.F.A., Zakaria S. (2022). Mechanical Properties and Water Absorption Capacity of Hybrid GFRP Composites. Polymers.

[22] Zhou Z.L., Yang Y.D. (1985). A preliminary discussion of creep mechanism of fiber reinforced plastics. FRP/Compos.

[23] Ghita C., Pop N., Popescu I.N. (2012). Existence result of an effective stress for an isotropic visco-plastic composite. Comp. Mater. Sci..

[24] Bai X.Y. (2015). Experimental Study and Theoretical Analysis of Anchoring Mechanism of GFRP Anti-Floating Anchors.

[25] Mu X.Y. (1990). Creeping Mechanics.

[26] Shen S.Z. (1982). Creep properties of FRP. Q. J. Mech..

[27] Dutta P.K., Hui D. (2000). Creep rupture of a GFRP composite at elevated temperatures. Comput. Struct..

[28] Tannous F.E., Saadatmanesh H. (1999). Durability of AR glass fiber reinforced plastic bars. J. Compos. Constr..

[29] Uomoto T., Nishimura T. (1999). Deterioration of aramid, glass, and carbon fibers due to alkali, acid, and water in different temperatures. ACI Spec. Publ..

[30] Li G.W., Gao L., Huang Z.H., Liu C.Q., Zhang D. (2007). Experiments on the pull-out model of damage mechanism of full-length bonded glass fiber reinforced polymer anchors. J. Rock. Mech. Geotech..

[31] Li G.W., Liu Z.Q., Huang Z.H., Cai Y.Q. (2010). Field test of applying glass fiber anchors to strengthen highway slopes. J. Rock. Mech. Eng..

[32] Sun Q. (2012). Experimental Study on the Mechanical Properties of GFRP Anchors under Freeze-Thaw Cycles and Long-Term Loading.

[33] Cui Y.H., Noruziaan B., Lee S., Chang M., Tao J. (2006). Glass fiber/wood plastic hybrid composites and their synergistic reinforcing effects. Polym. Mater. Sci. Eng..

[34] Guo D., Lu S.R., Luo C.X., Liu K., Ling R.H. (2012). Study on the structure and properties of glass fiber reinforced lignin/PP composites. Modern Plast. Process. Appl..

[35] Jeamtrakull S., Kositchaiyong A., Markpin T., Rosarpitak V., Sombatsompop N. (2012). Effects of wood constituents and content, and glass fiber reinforcement on wear behavior of wood/PVC composites. Compos. Part B Eng..

[36] Huang R., Xiong W., Xu X., Wu Q. (2012). Thermal expansion behavior of co-extruded WPCs with glass-fiber reinforced shells. Bioresources.

[37] Kim B.J., Huang R., Han J. (2014). Mechanical and morphological properties of coextruded wood plastic composites with glass fiber-filled shell. Polym. Compos..

[38] Zolfaghari A., Behravesh A.H., Adli A. (2013). Continuous glass fiber reinforced wood plastic composite in extrusion process: Mechanical properties. Mater. Des..

[39] Pulngern T., Udtaranakron T., Chanto K. (2020). Physical and Mechanical Behaviors of Thermally Modified Rubberwood Glulam Beam Under Sustained and Cyclic Loading. Wood Fiber Sci..

[40] Pulngern T., Chitsamran T., Chucheepsakul S., Rosarpitak V., Patcharaphun S., Sombatsompop N. (2016). Effect of temperature on mechanical properties and creep responses for wood/PVC composites. Constr. Build. Mater..

[41] Sain M.M., Balatinecz J., Law S. (2000). Creep fatigue in engineered wood fiber and plastic compositions. J. Appl. Polym. Sci..

[42] Li X.L., Liu W.Q., Fang H., Huo R.L., Wu P. (2019). Flexural creep behavior and life prediction of GFRP-balsa sandwich beams. Compos. Struct..

[43] Tian X.L., Li Z.G., Jiang Y.T., He Q., Wu C.Y. (2008). Study of creep properties of WPCs under different loading methods. Plast. Ind..

[44] Cao Y., Xu H.L., Wang W.H., Wang Q.W. (2016). Creep properties and creep modeling of molded poplar fiber/high density polyethylene composites. J. Compos..

[45] Du H.H., Wang W.H., Wang H.G., Wang Q.W. (2015). Effect of wood fiber content on creep properties of WPCs. J. Constr. Mater..

[46] Xie W.R. (2017). Design of Creep Testing Device and Creep Performance Study of WPCs.

[47] Cao J.Z. (2018). Wood Conservation and Modification.

[48] Lu X.C., Fang Q.H., Lu Q.Z., Chen F.Q., Sun X.M. (2009). Creep properties of PP/wood flour composites. Plastics.

[49] Jiang Y.T., Li Z.G., Wu Z.Y., Ding J.S. (2009). Creep and stress relaxation properties of WPCs. For. Mach. Woodwork. Equip..

[50] Shao X., He C.X., Jiang C.Y. (2019). Creep and thermal stability of wood fiber/PVC composites. J. Mater. Sci. Eng..

[51] Xu H.L., Cao Y., Wang W.H., Wang Q.W., Wang H.G. (2016). Effect of poplar fiber size on mechanical and creep properties of hot compression molded poplar fiber/high density polyethylene composites. J. Compos..

[52] Zhang X.L., Deng Z.C. (2022). Effects of Seawater Environment on the Degradation of GFRP Composites by Molecular Dynamics Method. Polymers.

[53] Corigliano A., Rizzi E., Papa E. (2000). Experimental characterization and numerical simulations of a syntactic-foam/glass-fibre composite sandwich. Compos. Sci. Technol..

[54] Daniel I.M., Gdoutos E.E. (2009). Failure Modes of Composite Sandwich Beams. Theor. Appl. Mech..

[55] George J., Sreekala M., Thomas S. (2001). A review on interface modification and characterization of natural fiber reinforced plastic composites. Polym. Eng. Sci..

[56] Sheng G.Y., Feng M.B. (2007). Engineering Materials Testing Technology.

[57] Hu J.H., Li Y.P., Chen W.J., Yang D.Q. (2020). A combined loading-creep model of ETFE foils for flat-patterning structures. Thin. Wall. Struct..

[58] Chen B., Guo L.P., Zhang W.X., Bai Y., Wang X.F. (2021). Compressive creep behavior of cellulose fiber reinforced concrete. IOP Conf. Ser. Earth Environ. Sci..

[59] Song Y.Q., Li X.L., Ma H.F., Fu H. (2021). Exploration of the full process of creep in generalized Kelvin model based on damaged body elements. Appl. Math. Mech..

[60] Shi T.B., Hu J.H., Chen W.J., Gao C.J. (2020). A refined numerical model for determining inflation-burst behavior of composite membrane structures. Polym. Test..

[61] Ye L.P., Feng P. (2006). Application and development of FRP in engineering structures. Chin. Civil. Eng. J..

[62] Li C.G., Guo R., Wang J.Q., Huang H.X., Xian G.J. (2021). Performance evolution of CFRP@GFRP hybrid composite rods under water immersion environment. J. Compos. Mater..

[63] Feng P. (2014). Development and application of composite materials in civil engineering. FRP/Compos..

[64] Cui X., Liu J., Xiao J.Y., Zhong Y. (2013). Research progress of vacuum introduction molding process. Mater. Guide.

[65] (2015). Standard Specification for Evaluation of Duration of Load and Creep Effects of Wood and Wood-Based Products.

